# Potential life-years gained over a 5-year period by correcting DOPPS-identified modifiable practices in haemodialysis: results from the European MONITOR-CKD5 study

**DOI:** 10.1186/s12882-019-1251-z

**Published:** 2019-03-05

**Authors:** Christian Combe, Johannes Mann, David Goldsmith, Frank Dellanna, Philippe Zaoui, Gérard London, Kris Denhaerynck, Andriy Krendyukov, Ivo Abraham, Karen MacDonald

**Affiliations:** 1 Centre Hospitalier Universitaire de Bordeaux and Unité INSERM 1026, University of Bordeaux, Bordeaux, France; 2 Friedrich Alexander Universität Erlangen-Nürnberg, Erlangen, Germany; 3 Guy’s and St Thomas’ NHS Foundation Hospital, London, UK; 4 DaVita Clinical Research Germany, Duesseldorf, Germany; 5 Université de Grenoble, Grenoble, France; 6 Centre Hospitalier F.H. Manhés, Fleury-Mérogis, France; 7 Matrix45, Tucson, AZ USA; 8 Department of Public Health, University of Basel, Basel, Switzerland; 9 Hexal AG, Holzkirchen, Germany; 10 University of Arizona College of Pharmacy and College of Medicine, Tucson, AZ USA

**Keywords:** Haemodialysis, Modifiable haemodialysis practices, Life-years, Biosimilar epoetin alfa, Anaemia

## Abstract

**Background:**

DOPPS reported that thousands of life-years could be gained in the US and Europe over 5 years by correcting six modifiable haemodialysis practices. We estimated potential life-years gained across 10 European countries using MONITOR-CKD5 study data.

**Methods:**

The DOPPS-based target ranges were used, except for haemoglobin due to label changes, as well as DOPPS-derived relative mortality risks. Percentages of MONITOR-CKD5 patients outside targets were calculated. Consistent with the DOPPS-based analyses, we extrapolated life-years gained for the MONITOR-CKD5 population over 5 years if all patients were within targets.

**Results:**

Bringing the 10 MONITOR-CKD5 countries’ dialysis populations into compliance on the six practices results in a 5-year gain of 97,428 patient-years. In descending order, survival impact was the highest for albumin levels, followed by phosphate levels, vascular access, haemoglobin, dialysis adequacy, and interdialytic weight gain.

**Conclusions:**

Optimal management of the six modifiable haemodialysis practices may achieve 6.2% increase in 5-year survival.

**Trial Registration:**

NCT01121237. Clinicaltrials.gov registration May 12, 2010 (retrospectively registered).

**Electronic supplementary material:**

The online version of this article (10.1186/s12882-019-1251-z) contains supplementary material, which is available to authorized users.

## Background

The Dialysis Outcomes and Practice Patterns Study (DOPPS) [1] reported [2] that an additional 143,617 patient life-years could be gained in the United States over a 5-year period if six modifiable haemodialysis practices were brought into best practice compliance. Four of the six modifiable haemodialysis practices (dialysis dose, phosphate control, improved anaemia, and partial correction of serum albumin) are supported by published guidelines [3–6] while reduced inter-dialytic weight gain [7, 8] and reduced use of catheters for vascular access [9–12] have been linked to mortality. These analyses were replicated for Belgium [13], Spain [14], France [15] and Sweden [16], except that albumin-corrected serum calcium was substituted for intradialytic weight gain. Here, we present a simulation of potential life-years gained by correcting these six practices across 10 European countries using data from the MONITOR-CKD5 study.

## Methods

MONITOR-CKD5, a real-world, prospective, observational study on the treatment patterns and associated outcomes of renal anaemia management with Sandoz epoetin alfa biosimilar (commercially available as Binocrit®/Epoetin Alfa Hexal®; hereafter Binocrit®), included an evaluable sample of 2023 CKD5 patients from 114 centres in 10 European countries: Austria (6 centres), France (35), Germany (45), Italy (21), Poland (7), Romania (14), Slovenia (2), Spain (6), Switzerland (4), and the United Kingdom (4). Eligible were male or female CKD5 adult (age ≥ 18) patients on haemodialysis of original or grafted kidneys; diagnosed with renal anaemia; and treated with intravenous Binocrit®. The overall study methodology has been detailed elsewhere [17]. The original DOPPS methodology to estimate life-years gained from modifiable haemodialysis practices [2], as well as the methodology of the four country replications [13–16] and the MONITOR-CKD5 analyses are summarized in Table 1.

**Table 1:** Methodology

Study	Country Countries Estimated	n (sample) for RR analysis	Method for RR	n (sample) for “outside of range” analysis	Population estimation source	Population estimation method	Calculation of attributable patient-years	Equation for calculating attributable patient-years^
Port et al. 2004 [2]	US	Random sample (*n* = 17,245) from all 7 DOPPS I countries (*N* = 52,905)	6 Cox proportional hazards models (one for each factor); a 7th Cox survival model was also used which adjusted for all six HD practices simultaneously. All models were adjusted for age, race, years of ESRD, country & 15 summary comorbid conditions (CAD, CHF, other cardiac disease, PVD, HTN, cerebrovascular disease, DM, lung disease, dyspnea, Hx cancer (active or inactive, excluding skin cancer), GI bleeding in past 12 months, neurologic disease, psychiatric disease, HIV/AIDS, and recurrent skin disease (including gangrene).	1914 prevalent HD patients from US DOPPS II	2001 US Renal Data System Annual Data Report	Estimation of the total US HD patient population in 2004 by extrapolation of 2001 data using a 6% annual growth rate	The expected gain in patient-years was calculated from the difference in the area under the 5-year survival curves between survival of the US haemodialysis population based on actual current death rates versus projected survival of the US haemodialysis population if all patients currently outside of the six practice guidelines were instead within them.	PY = [(N_0_/L) * FD] + [(N_1_/L) * (t – (FD/L))], Assumptions: • t = 5 years • L = 0.251 if all patients outside of the target ranges for the six HD practices are brought within these target ranges • L = 0.30 if there is no change from the current proportion of patients outside the six target ranges • N_0_ = 313,000 • N_1_ = 116,477
Jadoul et al. 2007 [13]	Belgium	> 20,000 (from all 12 DOPPS I and II countries)	Same as US study except: 1 modifiable practice differed (substituted albumin-corrected serum calcium for IDWG); did not adjust for dyspnea	538 prevalent HD patients from Belgian DOPPS II	Outpatient HD patient population reported in the 2001 Flemish-speaking and French-speaking registries	Estimation of the total Belgian HD patient population in 2006 by extrapolation of 2001 data using a 7.5% annual growth rate	Same as US but on Belgian data	Same as US except: • L = 0.113 if all patients outside of the target ranges for the six HD practices are brought within these target ranges • L = 0.22 if there is no change from the current proportion of patients outside the six target ranges • N_0_ = 6373 • N_1_ = 1591
Piera et al. 2007 [14]	Spain	> 20,000 (from all 12 DOPPS I and II countries)	Same as US study except: 1 modifiable practice differed (substituted albumin-corrected serum calcium for IDWG); did not adjust for dyspnea	613 from Spanish DOPPS II	Informe de la Sociedad Española de Nefrología de 2005	Estimation of the total Spanish HD patient population in 2006 by extrapolation of 2005 data using a 5.1% annual growth rate	Same as US but on Spanish data	Same as US except: • L = 0.099 if all patients outside of the target ranges for the six HD practices are brought within these target ranges • L = 0.170 if there is no change from the current proportion of patients outside the six target ranges • N_0_ = 20,920 • N_1_ = 4902
Canaud et al. 2008 [15]	France	> 20,000 (from all 12 DOPPS I and II countries)	Same as US study except: 1 modifiable practice differed (substituted albumin-corrected serum calcium for IDWG); did not adjust for dyspnea	532 from French DOPPS II	Bulletin Epidemiologique Hebdomadaire de 2005	Estimation of the total French HD patient population in 2006 by extrapolation of 2005 data using a 5.3% annual growth rate	Same as US but on French data	Same as US except: • L = 0.157 if all patients outside of the target ranges for the six HD practices are brought within these target ranges • L = 0.223 if there is no change from the current proportion of patients outside the six target ranges • N_0_ = 31,987 • N_1_ = 6070
Wikström et al. 2010 [16]	Sweden	> 20,000 (from all 12 DOPPS I and II countries)	Same as US study except: 1 modifiable practice differed (substituted albumin-corrected serum calcium for IDWG); did not adjust for dyspnea	547 prevalent HD patients from Swedish DOPPS II	2003 Swedish Renal Registry	Estimation of the total Swedish HD patient population in 2006 by extrapolation of 2003 data using a 4.5% annual growth rate	Same as US but on Swedish data	Same as US except: • L = 0.180 if all patients outside of the target ranges for the six HD practices are brought within these target ranges • L = 0.274 if there is no change from the current proportion of patients outside the six target ranges • N_0_ = 2434 • N_1_ = 769
MONITOR-CKD5	Austria France Germany Italy Poland Romania Slovenia Spain Switzerland United Kingdom*	Not applicable	Utilizes published RR rates from various DOPPS studies (see Table 2 for details)	2023 from MONITOR-CKD5	Austria, Poland, Romania, Spain, Switzerland, UK: ERA-EDTA Registry 2014; France: REIN Rapport Annuel 2014; Germany: QuaSi-Niere-Bericht 2006–2007; Italy: RIDT Report 2011–2013; Slovenia: ERA-EDTA Registry 2013	Estimation of the HD patient population in 2017 in the 10 countries participating in the MONITOR-CKD5 study by extrapolation of 2014 data (except for Germany for which 2006 data were available, and Italy and Slovenia for which 2013 data were available) using a 5% annual growth rate	Same as US but on data from 10 European countries	Same as US except: L = 0.170 if all patients outside the target ranges for the six HD practices are brought within these target range L = 0.203 if there is no change from the current proportion of patients outside the six target ranges N_0_ = 303,517 N_1_ = 66,597

* Included centres in England only.

^ where *t* total time period, *FD* fraction still on dialysis at time t, *L* annual rate of US haemodialysis patients lost from the haemodialysis population due to death or transfer to transplantation or peritoneal dialysis, *N*_0_ number of patients prevalent at the start of the 5-year time interval, *N*_1_ number of incident patients entering the haemodialysis patient population during each year, and *PY* projected total haemodialysis patient-years summed over the 5 years of the analysis period.

### Definition and Selection of Target Ranges for Modifiable Practices

We used the same target ranges set forth in the 2004 DOPPS analysis [2] for dialysis dose (Kt/V ≥ 1.2), phosphate control (serum phosphate ≤5.5 mg/dL), and fluid management (interdialytic weight gain ≤5.7%). For partial correction of serum albumin, we applied the range used in the Belgian [13], Spanish [14], and French [15] replications (≥4.0 g/dL). Best practice guidelines for anaemia management have changed since 2004 with more conservative haemoglobin targeting and individualized risk-based dosing of erythropoiesis-stimulating agents [5, 18, 19]. Hence, the original [2] haemoglobin target of ≥11 g/dL was reduced to ≥10 g/dL in our analysis. For the evaluation of catheter use for vascular access, all DOPPS-based analyses [2, 13–16] used a facility-level variable (percentage of catheter use at the centre-level). In the MONITOR-CKD5 study, vascular access was not collected as a facility-level but as a dichotomous patient-level variable. Some centres contributed small numbers of patients, hence determining centre percentages would have been subject to bias.

### Estimation of “Out of Range”

Table 2 presents the recommended targets for each modifiable factor for the original DOPPS study [2] and the replication studies [13–16] and the percentage of each study sample outside the target range. We calculated the valid percentages of patients outside the indicated range for the MONITOR-CKD5 evaluable sample (*n* = 2023).

**Table 2:** Modifiable practices: target ranges, percent of patients outside recommended range and associated relative risk of mortality

Country	Kt/V			Phosphate			Hb			Inter-Dialytic Weight Gain			Albumin			Facility Catheter Use			All 6 Factors		
	Target range	% outside range	RR if outside range	Target Range	% outside range	RR if outside range	Target Range	% outside range	RR if outside range	Target Range	% outside range	RR if outside range	Target Range	% outside range	RR if outside range	Target Range	% outside range	RR if outside range	% inside range on all 6	% outside range on ≥1	RR if outside range on ≥1
US[2]	≥1.2	12.1%	1.16	≤5.5 mg/dL	49.2%	1.11	≥11 g/dL	27.2%	1.14	≤5.7%	12.5%	1.22	≥3.5 g/dL	20.5%	1.38	≤7%	50.0%	1.23	8.7%	91.3%	1.33
Belgium [13]	≥1.2	30.3%	1.13	≤1.5 mmol/L	56.2%	1.11	≥11 g/dL	33.6%	1.20				≥4.0 g/dL	67.1%	1.46	≤10%	91.1%	1.20			
Spain [14]	≥1.2	22.7%	1.13	≤1.5 mmol/L	63.5%	1.11	≥11 g/dL	30.4%	1.20				≥4.0 g/dL	71.4%	1.46	≤10%	42.8%	1.20			
France [15]	≥1.2	15.5%	1.13	≤1.8 mmol/L	39.6%	1.14	≥11 g/dL	40.5%	1.20				≥4.0 g/dL	70.5%	1.46	≤10%	33.4%	1.20			
Sweden [16]	≥1.2	20.1%	1.13	≤1.8 mmol/L	50.8%	1.14	≥11 g/dL	23.8%	1.20				≥3.5 g/dL	40.1%	1.48	≤10%	88.2%	1.20			
MONITOR-CKD5 *	≥1.2	17.1%	1.13	≤5.5 mg/dL	38.5%	1.14	≥10 g/dL	14.0%	1.22	≤5.7%	7.8%	1.22	≥4.0 g/dL	56.8%	1.46	Pt-level: fistula (native or synthetic) vs. catheter	16.6%	1.32	15.5%	84.5%	1.33
Source of RR for MONITOR-CKD5	Four European replications: Jadoul et al. 2007 [Ref 13], Piera et al. 2007 [Ref 14], Canaud et al. 2008 [Ref 15], Wikström et al. 2010 [Ref 16]			Two European replications: Canaud et al. 2008 [Ref 15], Wikström et al. 2010 [Ref 16]			Locatelli et al. 2004 [Ref 20]			Port et al. 2004 [Ref 2]			Three European replications: Jadoul et al. 2007 [Ref 13], Piera et al. 2007 [Ref 14], Canaud et al. 2008 [Ref 15]			Pisoni et al. 2009 [Ref 21]			RR if outside range on ≥1 modifiable factor extrapolated from Port et al. 2004 [Ref. 1] Tables 1 and 2 where RR = (overall mortality / mortality rate of patients outside of range) / % of patients outside range − % of patients inside range; % of patients inside range on all 6 factors obtained through personal communication with Arbor Research 23Sep2016		

* Valid % reported

*Hb* haemoglobin, *RR* relative risk

### Selection of Mortality Relative Risk Values

In MONITOR-CKD5 patients were followed for up to 24 months, which is shorter than in DOPPS. Per expert opinion, except for haemoglobin and catheter use, we adopted the mortality RR values generated by the five country-specific analyses (Table 2) [2, 13–16] based on congruence in definition of target range between the MONITOR-CKD5 and these studies, and similarities in population. Due to guideline-recommended changes in Hb targets since 2004, the RR of 1.22 associated with Hb < 10 g/dL reported by Locatelli et al. was utilized [20]. This is an estimate adjusted for age, gender, comorbidities, mobility, malnourishment, ability to eat independently, years with end-stage renal disease (ESRD), country, and facility. Since MONITOR-CKD5 study measured catheter use at the patient level, the mortality RR adopted for this parameter was based on findings from a patient-level analysis of mortality risk associated with vascular access from DOPPS [21]; this estimate was adjusted for age, gender, race, years with ESRD, body weight, and comorbidities to reduce treatment-by-indication bias.

### Population Estimation

Haemodialysis prevalent and incident counts for the 10 countries in MONITOR-CKD5 are presented in Table 3. Data for 7 of the 10 countries were obtained from the 2014 ERA-EDTA Registry Annual Report [22] Slovenia was included in the 2013 [23] but not the 2014 ERA-EDTA Report; hence, 2013 data were utilized. Neither Germany nor Italy are included in the ERA-EDTA Registry. The most recent and sufficiently detailed data from their respective national databases were used instead, which were 2006 data for Germany [24] and 2013 data for Italy [25]. Country-level prevalent and incident counts were extrapolated from year of data to 2017 using an annual growth rate of 5%. However, this growth rate was not included in the calculation of projected life-years over the 5 years, thus providing a conservative, lower-bound estimation of life-years gained.

**Table 3:** Haemodialysis prevalent and incident counts

Countries	Source	Year of Data	HD Prevalent Count	HD Incident Count	Extrapolation to 2017	
					HD Prevalent Count	HD Incident Count
Austria	ERA-EDTA Registry 2014^1^	2014	3973	814	4599.24	942.31
France °	ERA-EDTA Registry 2014^1^	2014	41,314	8321	47,826.12	9632.60
Germany	Nierenersatztherapie in Deutschland 2006–2007^2^	2006	63,413	16,260	108,457.11	27,809.96
Italy	Registro Italiano di Dialisi e Trapianto Report 2011–2013^3^	2013	41,006	8241	49,843.22	10,017.40
Poland *†*	ERA-EDTA Registry 2014^1^ (Section B, aggregated data)^4^	2014	19,345	3954	22,394.26	4577.25
Romania ^	ERA-EDTA Registry 2014^1^	2014	14,686	2538	17,000.88	2938.05
Slovenia *††*	ERA-EDTA Registry 2013^5^	2013	1349	209	1639.72	254.04
Spain ~	ERA-EDTA Registry 2014^1^	2014	21,517	4236	24,908.62	4903.70
Switzerland	ERA-EDTA Registry 2014^1^ (Section B, aggregated data)	2014	2636	663	3051.50	767.51
United Kingdom, England only ° *	ERA-EDTA Registry 2014^1^	2014	20,556	4107	23,796.14	4754.37

° Incident counts are estimated (see ERA-EDTA Registry: Annual Report 2014 for methods[22])

*†* Incidence data for Poland not reported; incident count imputed

^ Overall prevalence of RRT is underestimated by approximately 3% due to an estimated 30% underreporting of patients living on a functioning graft

*††* Slovenia not included in ERA-EDTA Registry 2014, so 2013 ERA-EDTA data used[23]

~ Counts for Spain are sums of the following:

**Table:** 

Spain, Andalusia	4115	795
Spain, Aragon °	529	105
Spain, Asturias °	447	93
Spain, Basque country	789	159
Spain, Cantabria *	196	43
Spain, Castile & León °*	1078	217
Spain, Castile-La Mancha *	826	196
Spain, Catalonia °	4227	889
Spain, Extremadura °	590	106
Spain, Galicia	1540	292
Spain, Community of Madrid	2576	593
Spain, Region of Murcia	950	158
Spain, Navarre °*	251	61
Spain, Valencian region	3403	529

* Patients younger than 20 years of age are not reported

^1^ ERA-EDTA Registry: Annual Report 2014 [Ref 22]

^2^ Computed from 2006 dialysis prevalence (808 pmp X 95.2%HD) and incidence (213 pmp X 92.6%HD) rates from Nierenersatztherapie in Deutschland: Bericht über Dialysebehandlung und Nierentransplantation in Deutschland 2006-2007 [Ref 24] and 2006 population data (82,437,995) from Eurostat (http://ec.europa.eu/eurostat/)

^3^ Computed from 2013 dialysis prevalence (760 pmp X 90.4%HD) and incidence (160 pmp X 86.3%HD) rates from Registro Italiano Dialisi e Trapianto: Report 2011-2013 [Ref 25] and 2013 population data (59,685,227) from Eurostat (http://ec.europa.eu/eurostat/)

^4^ Prevalent count from ERA-EDTA Registry 2014¹ (Section B, aggregated data), incidence not reported; incident count imputed as Poland's HD prevalent count [19,345] X average percentage of HD incident count/HD prevalent count of 9 countries with complete data [0.2044]

^5^ ERA-EDTA Registry: Annual Report 2013 [Ref 23]

* Patients younger than 20 years of age are not reported

^1^ ERA-EDTA Registry: Annual Report 2014 [22]

^2^ Computed from 2006 dialysis prevalence (808 pmp X 95.2%HD) and incidence (213 pmp X 92.6%HD) rates from Nierenersatztherapie in Deutschland: Bericht über Dialysebehandlung und Nierentransplantation in Deutschland 2006–2007 [24] and 2006 population data (82,437,995) from Eurostat (http://ec.europa.eu/eurostat/)

^3^ Computed from 2013 dialysis prevalence (760 pmp X 90.4%HD) and incidence (160 pmp X 86.3%HD) rates from Registro Italiano Dialisi e Trapianto: Report 2011–2013 [25] and 2013 population data (59,685,227) from Eurostat (http://ec.europa.eu/eurostat/)

^4^ Prevalent count from ERA-EDTA Registry 2014^1^ (Section B, aggregated data), incidence not reported; incident count imputed as Poland’s HD prevalent count [19,345] X average percentage of HD incident count/HD prevalent count of 9 countries with complete data [0.2044]

^5^ ERA-EDTA Registry: Annual Report 2013 [23]

### Calculation of Attributable Patient-Years

We replicated the Port et al. [2] methodology for estimating potential life-years gained using the MONITOR-CKD5 proportions of patients out of range for each practice, the current population statistics for the 10 countries in the study, and the published relative risks associated with each practice. Based on the estimated percentages of patients falling outside the target ranges of the six modifiable practices and the mortality risk associated with each, Port el al. extrapolated to the US haemodialysis population for a 5-year period to quantify potential life-years gained [2].

Projections were obtained by computing the difference in the area under the 5-year survival curve between the general survival of haemodialysis patients based on the actual mortality rate and the estimated survival if patients outside targets were brought into target range on the six modifiable factors based. The projected total haemodialysis patient-years summed over the 5 years of the analysis period *PY* is defined as.

*PY = [(N*_*0*_/L)_*_ *FD]* + *[(N*_*1*_/L)_*_ *(t*–*(FD/L))]* (Eq. 1).

where *t* is the total time period in years, *FD* the fraction still on dialysis at *t*, *L* the annual rate of haemodialysis patients lost from the haemodialysis population due to death or transfer to transplantation or peritoneal dialysis, *N*_*0*_ the number of patients prevalent at the start of the 5-year time interval, and *N*_*1*_ the number of incident patients entering the haemodialysis population during each year. *PY* is calculated for status quo (*PY*_*0*_), where none of the patients outside the target range are brought into compliance; for each modifiable factor *i* assuming all patients are brought into compliance (*PY*_*i*_ where *i =* Kt/V, phosphate, Hb, interdialytic weight gain, albumin, AVF/graft); and for the condition that all patients are brought into compliance on all six factors (*PY*_*a*_).

From Eq. 1, we estimated the potential patient-years gained over the 5-year time interval *∆PY*_*5*_, for a given modifiable factor and aggregated across all six factors, as the difference between *PY*_*0*_ (the projected total patient-years summed over 5 years) and the corresponding *PY*_*i*_ for the *i*th factor or *PY*_*a*_ for the six factors aggregated; or.

*∆PY*_*5,i*_ *= PY*_*0*_ *- PY*_*i*_ (Eq. 2).

and

*∆PY*_*5,a*_ *= PY*_*0*_ *- PY*_*a*_ (Eq. 3).

### Ethics

The MONITOR-CKD5 study protocol was approved by the ethical review committees of participating centres in accordance with national laws and regulations. Written informed consent was obtained from all participating patients.

### Consort

N/A; this is not a clinical trial but a simulation study.

## Results

### Patients

A total of 2086 patients were enrolled yielding an evaluable enrolment sample of 2023 patients. Details of patient enrolment and disposition have been reported elsewhere [26] as have been centre-level and interim patient-level results [27]. Most patients (40.6%) were treated in Germany, followed by Romania (18.6%) and Italy (15.3%), accounting together for 74.4% of the study population. The sample was predominantly male (59.3%), virtually completely Caucasian (96.2%), with median age of 68 years (range 20–93). Median time on dialysis was 2.1 years (range 0–35). Most patients (82.5%) had been treated with another ESA at enrolment, therefore can be considered in the maintenance phase of renal anaemia management, and were switched to Binocrit®. Mean (±SD) Hb at enrolment was 11.09(±1.14)g/dL and 68.0% of patients had Hb values in the 10-12 g/dL range.

In terms of the six modifiable factors, as can be calculated from Tables 2, 82.9% were within target range for Kt/V (≥1.2), 61.5% had adequate phosphate control (serum phosphate ≤5.5 mg/dL), 86.0% had Hb ≥ 10 g/dL, 92.2% had interdialytic weight gain ≤5.7, 43.2% had partial correction of serum albumin (≥4.0 g/dL), and 83.4% had vascular access via either an arteriovenous fistula or graft.

### Estimated Patient Life-Years Gained

Table 4 summarizes the patient life-years gained over five years, estimated per Eqs. 1 through 3 above, if respectively an optimal 100% (or suboptimal 50%) of patients falling outside of the recommended target range on the six modifiable practices were brought into compliance. Results are reported for each modifiable factor separately, as well as for all 6 factors combined. The baseline for the 10 MONITOR-CKD5 countries is the estimated 303,517 haemodialysis patients for 2017. This baseline is adjusted upward for the estimated 66,597 incident cases added over the 5-year period, adjusted downward for the variable annual death rate in general and associated with compliance with each factor, and further adjusted downward for the fixed annual loss rate to peritoneal dialysis or transplant.

**Table 4:** Project patient-years gained based on relative risk for compliance with recommended targets (over 5 years)

Measure	Current statistics	Kt/V ≥ 1.2	Phosphate ≤ 5.5 mg/dL	Hb ≥ 10 g/dL	IDWG ≤ 5.7%	Albumin ≥ 4.0 g/dl	AVF or graft v. catheter	All 6 Factors
Annual death rate	0.150 ^1^	0.1467 ^2^	0.1423 ^2^	0.1455 ^2^	0.1475 ^2^	0.1189 ^2^	0.1424 ^2^	0.1173 ^2^
Annual rate of other loss^3^	0.053	0.053	0.053	0.053	0.053	0.053	0.053	0.053
L (Total loss rate)	0.203	0.200	0.195	0.199	0.200	0.172	0.195	0.170
T	5	5	5	5	5	5	5	5
N_0_^4^	303,517	303,517	303,517	303,517	303,517	303,517	303,517	303,517
N_1_^5^	66,597	66,597	66,597	66,597	66,597	66,597	66,597	66,597
FD	0.63760	0.63164	0.62343	0.62938	0.63298	0.57668	0.62363	0.57321
PY	1,563,221	1,572,540	1,585,273	1,576,047	1,570,443	1,655,565	1,584,967	1,660,650
Potential patient years gained								
if 100% brought within target		9319	22,052	12,826	7222	92,344	21,746	97,429^6^
if 50% brought within target^7^		4501	10,651	6195	3488	44,601	10,503	47,057

*Hb* haemoglobin, *IDWG* inter-dialytic weight gain, *AVF* arterio-venous fistula, *L* annual rate of US haemodialysis patients lost from the haemodialysis population due to death or transfer to transplantation or peritoneal dialysis, *t* total time period, *N*_*0*_ number of patients prevalent at the start of the 5-year time interval, *N*_*1*_ number of incident patients entering the haemodialysis patient population during each year, *FD* fraction still on dialysis at time t, *PY* projected total haemodialysis patient years summed over the 5 years of the analysis period

^1^ From ERA-EDTA Registry: Annual Report 2014. Table B.6.7.a. Unadjusted 1-year survival (cohort 2008–2012): incident dialysis patients (from day 91).

^2^ Mortality rate of pts. inside range = overall mortality / ((% of patients outside range * RR) + % of patients in range)

^3^ Other loss to peritoneal dialysis or transplant assumed to be 5.3%

^4^ N_0_ assumed to be 303,517 based upon population estimates for countries participating in MONITOR-CKD5 [Table 3]

^5^ N_1_ assumed to be 66,597 based upon population estimates for countries participating in MONITOR-CKD5 [Table 3]

^6^ Patient-years gained if all 6 factors in target range is less than the sum of the years gained on the 6 individual factors since the modifiable practices are not mutually exclusive (i.e. patients can be outside target range on ≥1 factor)

^7^ Adjusted to 0.4830 of potential years gained if 100% brought within target (i.e. not .50, due to results of sensitivity analysis; personal communication with Arbor Research)

The estimated potential patient-years gained over the 5-year period at status quo (*PY*_*0*_) is 1,563,221. At the singular modifiable factor level, bringing all patients into Kt/V compliance yields 9319 additional patient-years over *PY*_*0*_ (for a total of 1,572,540); into phosphate compliance an additional 22,052 patient-years (1,585,273); into Hb compliance an additional 12,826 patient-years (1,576,047); into interdialytic weight gain compliance an additional 7222 patient-years (1,570,443); into albumin compliance an additional 92,344 patient-years (1,655,565); and into vascular access compliance an additional 21,746 patient-years (1,584,967). On the aggregate, bringing all patients into compliance on all six modifiable factors results in an incremental gain of 97,428 patient-years for a total of 1,660,650 patient-years (note that years gained if all 6 factors in target range is less than the sum of the years gained on the 6 individual factors since the modifiable practices are not mutually exclusive; i.e. patients can be outside target range on ≥1 factor).

## Discussion

Using as baseline the estimated 303,517 haemodialysis patients estimated to be alive in 2017 in the 10 European countries that participated in the MONITOR-CKD5 study, and adjusting this upward for growth in this patient population and downward for mortality and transition to peritoneal dialysis or transplantation, we projected that the 5-year period starting in 2017 would yield a net total of 1,563,221 patient-years under the haemodialysis practice patterns observed in the study. However, as the DOPPS investigators have shown for several countries [2, 13–16], mortality in haemodialysis patients could potentially be reduced and life expectancy increased by focusing on six modifiable factors: dialysis quality (Kt/V), managing phosphate, haemoglobin, and albumin levels, reducing interdialytic weight gain (or, alternately managing calcium), and minimizing catheter use for vascular access.

If all haemodialysis patients in the 10 MONITOR-CKD5 countries were brought into the target range of these six modifiable practice patterns, an additional 97,429 patient life-years would hypothetically be gained over the 5-year period starting in 2017. This corresponds to a 6.2% increase in 5-year survival. In comparison, the corresponding rates from the country-specific DOPPS analyses of the modifiable practices are, in ascending order, 8.2% for the US, 13.1% for France, 14.7% for Spain, 17.8% for Sweden, and 22.5% for Belgium [2, 13–16]. The differences in rates may be due to sample size. However, considering that these simulation analyses cover a time span of approximately 15 years, this variation in rates may also suggest significant improvements having been achieved in haemodialysis care over time [28]. Though we chose not to perform country-specific analyses, with collectively 25.3% of MONITOR-CKD5 patients being treated in Central and Eastern European countries, our lower rate in life-years gained underscores the advances in the quality of haemodialysis care made across all European regions.

This is also evident from the loss rates used in the various analyses. Consistent with their time period and country, Port et al. [2] assumed a 24.0% annual death rate and 6.5% annual rate of other loss (transition or peritoneal analysis or transplantation), for a total loss rate of 30.5%. In the four country-specific DOPPS analyses [13–16], the annual death rates ranged from 17.1 to 24.3%, the annual other loss rates from a gain of 2.1% to a loss of 5.3%, for total loss rates between 17.0 and 27.4% In our ten-country European analysis, these rates were 15.0% (death-related), 5.3% (other), and 20.3% (total).

With the historical trends of fewer patients outside of the recommended range on each of the modifiable practices and decreasing mortality rates in dialysis populations, there is a diminishing opportunity for further improvement and, hence, smaller gains in potential life years. Yet, there is still room for improvement. While the percentage of patients out of range have decreased across all six modifiable practices, only 15% of patients in the MONITOR-CKD5 study were in range on all six.

The historical differences are also evident at the level of the individual practice patterns. In the Port et al. [2] analysis, reducing catheter use was associated with the greatest gain in patient life-years (with the highest potential gains in the USA), followed by increasing serum albumin levels (a measure of nutritional status and inflammation), and phosphate control. In our analysis, the greatest gains in theoretical patient life-years were achieved by targeting patient’s serum albumin level followed, remotely, by phosphate control and vascular access. In the country-specific DOPPS analyses (which did not include interdialytic weight gain but serum calcium level instead), albumin levels and catheter use were the modifiable factors that prevailed in impact, followed by calcium levels and, variably, phosphate and Hb levels. Despite these differences in rates, all analyses converged on the importance of bringing albumin levels into target range, assuring vascular access by means of fistula or graft, and mineral control. That dialysis quality contributed only modestly to survival in all analyses may reflect the longstanding acceptance of the Kt/V ≥ 1.2 standard. One notable challenge for clinicians is that while serum albumin exerts the most influence on mortality in these models in terms of relative risk, it is perhaps the most resistant of the six practices to therapeutic intervention, since it is linked to different factors, mainly inflammation and nutritional status [29].

Mortality risks attributable to the six modifiable practice patterns have not been systematically evaluated in randomized controlled trials (RCT); though a RCT of an established practice such as dialysis quality of Kt/V ≥ 1.2 versus < 1.2 would be ethically untestable. Hence, estimating preventable mortality and associated life-years gained as a function of modifiable practices may be appropriate as secondary endpoints in randomized trials. Further, the role of patient adherence, in particular to dietary restrictions, phosphate binders, and dialysis regimen, should be examined in future studies [30–32].

Our analyses have limitations. Like DOPPS, MONITOR-CKD5 is a non-controlled study. The use of patient-level versus facility-level data for catheter use may introduce treatment-by-indication bias especially in terms of patient age, comorbid conditions, and disease severity. The adjusted mortality risk associated with patient-level vascular access data utilized in our study [21] reduced such confounding but some residual bias cannot be excluded. Countries in MONITOR-CKD5 differed in some of the modifiable practices MONITOR-CKD5 [28]. The estimation of life-years gained using the MONITOR-CKD5 aggregate sample may conceal specific countries where opportunities for larger gains exist. Not being part of the ERA-EDTA Registry, national reports were used for Germany and Italy. The MONITOR-CKD5 patient population was virtually Caucasian with minimal racial/ethnic variation.

## Conclusions

Replicating and extending the DOPPS analyses [2, 13–16] to the population in MONITOR-CKD5, we estimated in this hypothetical simulation that bringing all haemodialysis patients of the countries included in this study within the target range of six modifiable practices would yield a 6.2% increase in patient life-years over 5 years. An additional 97,429 life-years might be gained if, in descending order of survival impact, haemodialysis patients could be maintained at albumin levels ≥4.0 g/dL and phosphate levels ≤5.5 mg/dL, have vascular access by means of fistula or graft, have haemoglobin maintained at ≥10 g/dL, receive dialysis at Kt/V ≥ 1.2, and have their interdialytic weight gain limited to ≤5.7%. It is reassuring that compared to the original DOPPS papers, potential gains linked to quality improvement have decreased, reflecting better achievement of recommended therapeutic goals.

## Additional file

Additional file 1: Supplementary List of all Ethical Review Committees for MONITOR-CKD5. (DOCX 99 kb)

## Abbreviations

AVF: Arteriovenous fistula
DOPPS: Dialysis Outcomes and Practice Patterns Study.
ESRD: End-stage renal disease.
Hb: Hemoglobin.
RR: Relative risk.
RCT: Randomized controlled trial.
SD: Standard deviation.
