# Supplementary material for: Potential life-years gained over a 5-year period by correcting DOPPS-identified modifiable practices in haemodialysis: results from the European MONITOR-CKD5 study
Source: BMC Nephrol. 2019 Mar 5;20:81. doi: 10.1186/s12882-019-1251-z (PMC6402099; doi:10.1186/s12882-019-1251-z)
Supplement: Supplementary file 1 — Supplementary List of all Ethical Review Committees for MONITOR-CKD5. (DOCX 99 kb) [file 12882_2019_1251_MOESM1_ESM.docx]

| **Supplementary List of all Ethical Review Committees for MONITOR-CKD5** |
| --- |
| France |
| Comité consultatif sur le traitement de l’information en matière de recherche dans le domaine de la santé Ministère de l'Enseignement supérieur et de la Recherche à l’attention de la Secrétaire générale Direction de la recherche et de l’innovation 1, rue Descartes - 75231 Paris Cedex 05 |
| Conseil National de l'Ordre des Médecins 180 Boulevard Haussmann 75008 Paris |
| Commission Nationale de l’Informatique et des Libertés 8 rue Vivienne CS 30223 75083 Paris Cedex 02 |
| Germany |
| Ethikkommission der Ärztekammer Sachsen-Anhalt Am Kirchtor 9 06108 Halle (Saale) |
| Bundesinstitut für Arzneimittel und Medizinprodukte Abteilung Arzneimittelsicherheit / Pharmakovigilanz Kurt-Georg-Kiesinger-Allee 3 D-53175 Bonn |
| GKV-Spitzenverband  Abteilung Arznei- und Heilmittel  Mittelstraße 51 10117 Berlin |
| Kassenärztliche Bundesvereinigung Geschäftsbereich Ärztliche und veranlasste Leistungen Abteilung Arzneimittel Herbert-Lewin-Platz 2 10623 Berlin |
| EK der Ärztekammer Nordrhein Tersteegenstr. 9 40474 Düsseldorf |
| Ethikkommission der Landesärztekammer Baden-Württemberg  Jahnstr. 40 70597 Stuttgart |
| Ethik-Kommission der Bayerischen Landesärztekammer Mühlbaurstraße 16 81677 München |
| Landesärztekammer Brandenburg Dreifertstraße 12 03044 Cottbus |
| Ethikkommission an der Medizinischen Fakultät Ernst-Moritz-Arndt-Universität Greifswald Institut für Pharmakologie Friedrich-Loeffler-Str. 23 d 17487 Greifswald |
| Dr. Silke Schrum Humboldtstr. 67a 22083 Hamburg |
| EK der Landesärztekammer Hessen Im Vogelsgesang 3 60446 Frankfurt |
| Ethikkommission zur Beurteilung medizinischer Forschung am Menschen Berliner Allee 20 30175 Hannover |
| Ethikkommission der Landesärztekammer Rheinland-Pfalz Deutschhausplatz 3 55116 Mainz |
| Ethikkommission an der Medizinischen  Fakultät der Universität Rostock St.-Georg-Str. 108 18055 Rostock |
| Ethikkommisson bei der Sächsischen Landesärztekammer Schützenhöhe 16 01099 Dresden |
| Ethik-Kommission bei der Ärztekammer Schleswig-Holstein Bismarckallee 8-12 23795 Bad Segeberg |
| Landesärztekammer Thüringen Ethikkommission Im Semmicht 33 07751 Jena |
| Ethik-Kommission der Ärztekammer Westfalen-Lippe und der Medizinischen Fakultät der Westfälischen Wilhelms-Universität Münster Gartenstr. 210 - 214 D-48147 Münster |
| Italy |
| Comitato Etico Dell`Azienda Ospedaliero-Universitaria  Policlinico-Vittorio Emanuele di Catania Policlinico “G. Rodolico” - Via S. Sofia, 78  95123 Catania |
| Comitato Etico Della ASL BA di Bari Lungomare Starita n. 6   70123 Bari |
| Comitato Etico Della ASL TO/2 di Torino corso Svizzera 185 BIS  10149 Torino |
| Comitato Etico Della ASL di Foggia Piazza Liberta´ 1  71100 Foggia |
| Comitato Etico Della ASL 3 Genovese di Genova Via Bertani 4  16125 Genova |
| Comitato Etico Dell'Azienda Sanitaria Provinciale di Catania  Via S. Maria la Grande, 5  95124 Catania |
| Comitato Etico Dell'Azienda Ospedaliera Per L´Emergenza Cannizzaro di Catania  Via Messina 829  95126 Catania |
| Comitato Etico Centrale Dell`IRCCS Fondazione Salvatore Maugeri di Pavia Via Salvatore Maugeri, 4  27100 Pavia |
| Comitato Etico Dell'Azienda Ospedaliera S. Andrea di Roma Via di Grottarossa 1035-1039  00189 Roma |
| Comitato Etico Dell'Azienda Ospedaliera di Cosenza  Via S. Martino S.N.  87100 Cosenza |
| Comitato Etico Aziendale Della AUSL di Rieti Viale Matteucci, 9  02100 Rieti |
| Comitato Etico Dell`Universita`Cattolica del Sarco Cuore – Policlinico Universitario Agostino Gremelli di Roma  Largo A. Gemelli, 8  00168 Roma |
| Comitato Etico Sperimentazione Clinica Medicinali Della AUSL 8 di Arezzo  Via Curtatone 54  52100 Arezzo |
| Comitato Etico Dell'Azienda Ospedaliera Universitaria S. Giovanni Battista di Torino  C.so Bramante 88/90  10126 Torino |
| Comitato Etico Interaziendale Corso Mazzini n. 18 28100 Novara |
| Comitato Etico Dell'Azienda Ospedaliera S. Croce E Carle di Cueno  Via Monte Zovetto, 18  12100 Cuneo |
| Comitato Etico Dell'Azienda Ospedaliera Istituti Clinici di Perfezionamento di Milano Via Castelvetro 32 21054 Milano |
| Comitato Etico Della ASL di Frosinone  VIA A. FABI s.n.c.  03100 Frosinone |
| Comitato Etico Dell'Azienda Sanitaria Provinciale di Ragusa  Piazza Igea N. 1  97100 Ragusa |
| Comitato Etico Dell'Azienda Ospedaliera Universitaria S. Luigi Gonzaga di Orbassano Regione Gonzole 10  10043 Orbassano – TO |
| Poland |
| Niezależna Komisja Bioetyczna do Spraw Badań Naukowych przy GUMed. M. Skłodowskiej-Curie 3a 80-210, Gdańsk |
| Romania |
| Agentia Nationala a Medicamentului si Dispozitivelor Medicale Aviator Sanatescu street, 48, Sector 1, Bucuresti |
| Slovenia |
| Javna Agencija Republike Slovenije Za Zdravila in Medicinske  Pripomočke  Ptujska Ulica 21 1000 Ljubljana |
| Inštitut za klinično nevrofiziologijo, Klinični center Ljubljana Zaloška c. 7 1525 Ljubljana |
| Spain |
| Agencia Española del Medicamento y Productos Sanitarios C/Campezo 1, Edificio 8 28022 Madrid |
| Junta de Castilla y Leon Consejería de Sanidad Pseo. de Zorrilla, 1 - C.P 47007 - Valladolid (Valladolid) Castilla y León |
| Consejeria de Salud Secretaria General de Calidad y Modernizacion Comité Autonomica de Ensayos Clinicos Avd. Innovacion s/n. Edifico Arena 1 41020 Sevilla |
| CEIC Virgen del Rocio Avda. Manuel Siurot, s/n  41013 – Sevilla |
| CEIC Hospital Sara T. Saez Jimenez Subdirectora de Gestion y SS. GG. Complejo Asistencial de Ávila Avd. Juan Carlos l s/n. 05071 Avila |
| CEIC del Area de Salud de Zamora Hospital Virgen de la Concha Avda. Hernán Cortés, 40 49021 Zamora |
| CEIC Hospital Virgen de la Macarena Hospital Univ. “Virgen Macarena” y Centros de salud del Area Sanitaria Avda. Dr. Fedriani, 3 41009 – Sevilla |
| CEIC Secretaria del Dr. J. M. Culebras, Presidente del CEICL Complejo Asistencial Universitario de León Edificio "San Antonio Alad" 2a Planta - Cirugia General II Altos de Nava s/n. 24071 Leon |
| Switzerland |
| Swiss Medic Hallerstr. 7 3000 Bern 9 |
| VAUD Commission cantonale (VD) d'éthique de la recherche sur l'être humain Rue du Bugnon 21 CH-1011 Lausanne |
| Kontanale Ethikkommission Universitätshospital Zürich Sonneggstraße 12 8091 Zürich |
| Commission cantonale valaisanne d'éthique médicale (CCVEM) Avenue du Grand Champuc 86 ICHV 1950 Sion |
| Commission cantonale d'éthique de recherche Route des Arsenaux 16, Case Postale 1705 Fribourg |
| United Kingdom |
| Cambridgeshire 1 Research Ethics Commitee Victoria House Capital Park, Fulbourn Cambridge, CB21 5XB |
| R&D Cambridge University Hospitals NHS Foundation Trust Addenbrooke´s Hospital Hills Road Cambridge, CB2 0QQ |
| R&D Countess of Chester Hospital NHS Foundation Trust Research and Innovation Department The Countess of Chester Hospital Health Park Chester, Cheshire, CH2 1UL |
| Plymouth Hospitals NHS Foundation Trust R&D office, Room N17, ITTC Building Tamar Science Park Plymouth, Devon, PL6 8BX |
| R&D (JRO) Level 3 East Norfolk and Norwich University Hospitals NHS Foundation Trust Colney Lane Norwich, NR4 7UY |
